# “LONG COVID”—A hypothesis for understanding the biological basis and pharmacological treatment strategy

**DOI:** 10.1002/prp2.911

**Published:** 2022-01-13

**Authors:** Bevyn Jarrott, Richard Head, Kirsty G. Pringle, Eugenie R. Lumbers, Jennifer H. Martin

**Affiliations:** ^1^ Florey Institute of Neuroscience & Mental Health University of Melbourne Parkville Victoria Australia; ^2^ University of South Australia Adelaide South Australia Australia; ^3^ School of Biomedical Sciences and Pharmacy Faculty of Health and Medicine University of Newcastle Newcastle New South Wales Australia; ^4^ Centre for Drug Repurposing and Medicines Research Clinical Pharmacology University of Newcastle New Lambton New South Wales Australia

**Keywords:** “LONG COVID”, COVID‐19, endothelium, melatonin, NRF2, oxidative stress, SARS‐CoV‐2, tissue hypoxia

## Abstract

Infection of humans with SARS‐CoV‐2 virus causes a disease known colloquially as “COVID‐19” with symptoms ranging from asymptomatic to severe pneumonia. Initial pathology is due to the virus binding to the ACE‐2 protein on endothelial cells lining blood vessels and entering these cells in order to replicate. Viral replication causes oxidative stress due to elevated levels of reactive oxygen species. Many (~60%) of the infected people appear to have eliminated the virus from their body after 28 days and resume normal activity. However, a significant proportion (~40%) experience a variety of symptoms (loss of smell and/or taste, fatigue, cough, aching pain, “brain fog,” insomnia, shortness of breath, and tachycardia) after 12 weeks and are diagnosed with a syndrome named “LONG COVID.” Longitudinal clinical studies in a group of subjects who were infected with SARS‐CoV‐2 have been compared to a non‐infected matched group of subjects. A cohort of infected subjects can be identified by a battery of cytokine markers to have persistent, low level grade of inflammation and often self‐report two or more troubling symptoms. There is no drug that will relieve their symptoms effectively. It is hypothesized that drugs that activate the intracellular transcription factor, nuclear factor erythroid‐derived 2‐like 2 (NRF2) may increase the expression of enzymes to synthesize the intracellular antioxidant, glutathione that will quench free radicals causing oxidative stress. The hormone melatonin has been identified as an activator of NRF2 and a relatively safe chemical for most people to ingest chronically. Thus, it is an option for consideration of re‐purposing studies in “LONG COVID” subjects experiencing insomnia, depression, fatigue, and “brain fog” but not tachycardia. Appropriately designed clinical trials are required to evaluate melatonin.

## Graphical abstract

It is accepted that SARS‐CoV‐2 virus is transmitted in the air by exhaled droplets from infected people and if inhaled by a close contact, it binds to endothelial cells of blood vessels in the naso‐pharnygeal tract where it rapidly replicates. This causes oxidative stress due to formation of free radicals that inhibits the normal synthesis of cytoprotective enzymes and proteins resulting in prolonged depletion of the intracellular antioxidant, glutathione which could be a cause of “LONG COVID”. It is hypothesized that elevation in the levels of the key transcription factor NRF2 by the hormone, melatonin will restore enzymes that synthesize glutathione.

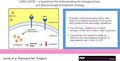

**Abbreviations**
ACE‐2: angiotensin‐converting enzyme‐2
COVID‐19: Coronavirus Disease 2019
Keap1: Kelch‐like ECH‐Associated Protein‐1
NFkB: nuclear factor kappa‐B
NICE: National Institute for Health and Care Excellence (UK)
NLRP3: nucleotide‐binding oligomerization domain‐like receptor containing pyrin domain 3
NRF2: nuclear factor, erythroid 2 like 2
ROS: reactive oxygen species
SARS‐CoV‐2: severe acute respiratory syndrome coronavirus 2

## 1 INTRODUCTION

In January 2020, a novel coronavirus was isolated from the respiratory tract of patients with atypical severe viral pneumonia in Wuhan, China and named severe acute respiratory syndrome corona virus 2 (SARS‐CoV‐2) by the World Health Organization on January 11. When its genome was sequenced, it was found to be a single‐strand RNA‐enveloped virus that was different from known corona viruses such as SARS‐CoV and MERS‐CoV. Its molecular properties have been reviewed in this Journal.^1^ The pathological responses to this viral infection range from asymptomatic to severe respiratory and multiple organ failures, primarily in adults, and is called COVID‐19. In a Viewpoint, Thomas Lüscher^2^ stated that the SARS‐CoV‐2 virus “produces protean manifestations ranging from head to toe, wreaking seemingly indiscriminate havoc on multiple organ systems including the lungs, heart, brain, kidney and the vasculature. This Viewpoint presents the hypothesis that COVID‐19, particularly in the later complicated stages, represents an endothelial disease. Cytokines, protein proinflammatory mediators, are key signals that shift endothelial function from the homeostatic into the defensive mode. The endgame of COVID‐19 involves a cytokine storm with positive feedback loops governing cytokine production that over‐whelm counter‐regulatory mechanisms.”

Tragically, SARS‐CoV‐2 has caused a pandemic that has resulted in around 14 million excess deaths and 220 million infections worldwide by September 2021. It is acknowledged that the prevalence rates of infection have been widely underestimated, including in countries with developed diseases notification systems. For example, a large study in the United States measured IgG and IgM antibodies against the SARS‐CoV‐2 spike protein and its receptor binding domain in finger‐tip blood obtained from 11 382 citizens with characteristics that reflected US population.^3^ It was found that there were 4.8 undiagnosed SARS‐CoV‐2 infections for every diagnosed case of COVID‐19 in this population sample which gives an estimate of 16.8 million undiagnosed infections in US population by mid‐July 2020.

## 2 ACTIONS OF SARS‐CoV‐2

It is now generally accepted that the virus spreads through a variety of routes including oral, but predominantly via the air in expired droplets and aerosols from humans. It can be inhaled by any others within 1.5 m (particularly when indoors) via the epithelial cells of the mucous membranes in the nose, eyes, and mouth.^4^ The virus enters cells that are expressing the plasma membrane enzyme, ACE‐2, and then it takes over the cells’ synthetic macromolecules resulting in rapid replication and shedding. Unfortunately, the infected person does not experience symptoms for 2–3 days when replication and shedding are high and they are said to be “asymptomatic spreaders”.^4^ Then immune cells (T cells, neutrophils, macrophages, mast cells) of the host attack infected cells leading to release of autacoids such as histamine, bradykinin, prostanoids, ATP, and cytokines that give symptoms such as cough, fever, headache, lethargy, rhinorrhea, anosmia (loss of smell), and ageusia (loss of taste). This prompts the host to get tested with the gold standard PCR assay to confirm infection with SARS‐CoV‐2.^4^ At the molecular level, SARS‐CoV‐2 infection within the cell hijacks mitochondrial function that leads to accumulation of mitochondrial DNA in the cytosol. This form of DNA induces inflammasome activation and suppression of innate and adaptive immunity.^5^ The nascent virus spreads further into the nasopharyngeal tract. As outlined by Head et al. in this Journal^37^ in many people, SARS‐CoV‐2 reaches the lungs causing an acute respiratory distress (ARDS) because the virus has a lethal action on infected alveoli that impairs oxygenation of blood. The virus then crosses the alveoli–blood barrier and because of the widespread cellular distribution of its target ACE‐2 results in other organs such as heart, liver, kidney, and CNS becoming infected, provoking an immune response and organ failure, covered in Lumbers et al.^32^ This can be fatal particularly in elderly adults with comorbidities such as hypertension, type 2 diabetes, and obesity.^4^ The United States among others have reported that young adults with obesity are more likely to require hospitalization and develop more severe disease than non‐obese young adults,^6^ consistent with the known increased distribution and activity of the renin–angiotensin system in obesity.^38^ For patients who survive, there is increasing documented incidences of up to 50% people with a lack of return of taste and smell sensations for at least 6 months, chronic fatigue, respiratory impairment, and behavioral effects described as “brain fog” or depression. By common usage, this has been described as “LONG COVID.”

## 3 WHAT IS “LONG COVID”?

The colloquial term “LONG COVID” is also known as Post‐Acute Sequelae of SARS‐CoV‐2 (PASC) or Post‐COVID‐Syndrome (PCS). The British National Institute for Health and Care Excellence (NICE) defines PCS as “signs and symptoms that develop during or after an infection consistent with Covid‐19, continue for more than 12 weeks and are not explained by an alternative diagnosis”.^7^ While a helpful definition, NICE does not list the signs and symptoms, likely because the standard nomenclature process has not yet been undertaken. The British Medical Journal convened an expert panel in September 2020 to more tightly define the syndrome as “not recovering for several weeks or months following the start of symptoms that were suggestive of Covid, whether you were tested or not”.^8^ Interestingly, a panel member commented that “a persistent cough, hoarse voice, headache, skipping meals and shortness of breath in the first week” meant that “you are two or three times more likely to get longer‐term symptoms.” The panel also said that the numbers of patients with “LONG COVID” symptoms should be included in the COVID‐19 statistics of positive test results and deaths.

Note that patients with severe COVID‐19 who were hospitalized in ICUs on mechanical ventilators and in prone position for long periods suffer consequences of this treatment lasting months and should not be considered to have “LONG COVID.” Similarly, some patients may have developed lung fibrosis that leads to dyspnea and persistent dry cough, also are not usually defined as fitting the definition of “LONG COVID.”

**FIGURE 1:**
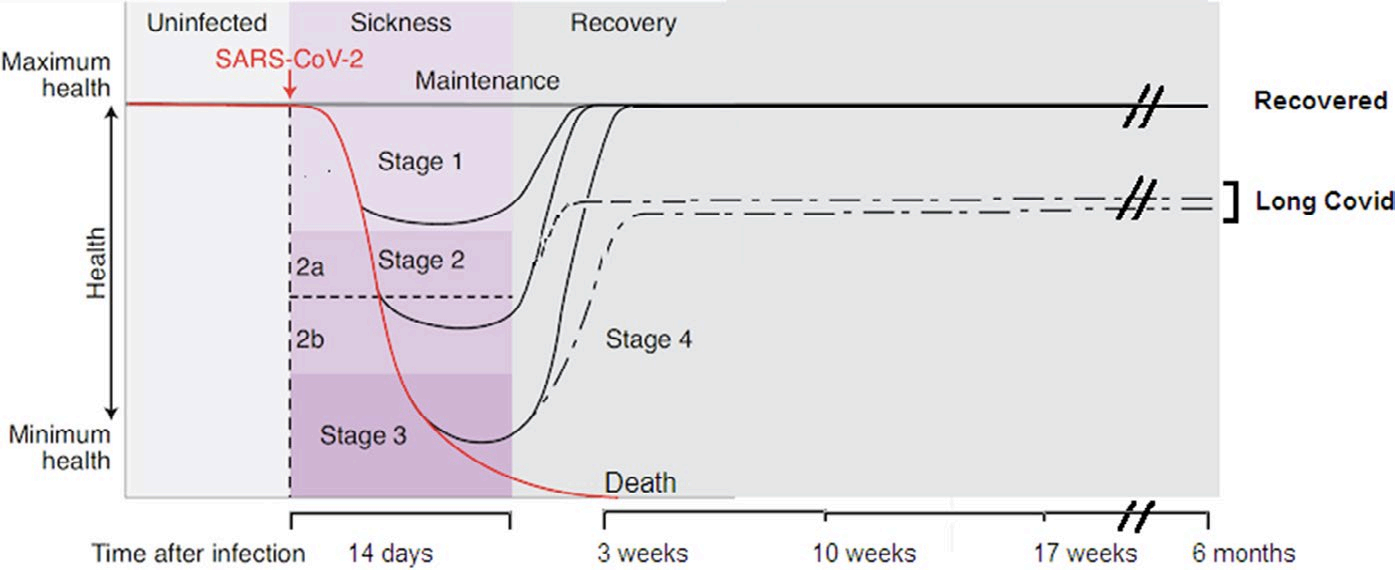
The disease phases of patients with COVID‐19, modified from Ayres.^11^ After infection (vertical dotted line), a proportion of patients (often young adults) can remain healthy and show no signs of sickness over the next 6 months. For patients who become symptomatic, the disease course can be described in four stages: Stage 1 is mild with patients exhibiting fever, malaise, and dry cough followed by full recovery. Stage 2 is characterized by a pneumonia phase without hypoxia (2a) or with hypoxia (2b) with some patients recovering fully and some with multiple symptoms (dotted line) that persist and are said to have “LONG COVID.” A proportion of patients progress to stage 3 when they develop acute respiratory syndrome, shock, or multi‐organ failure. Some will die by 3 weeks and some will recover either fully or enter stage 4 with partial recovery (dotted line) and have “LONG COVID”

It is accepted that at this time in “LONG COVID” patients, the virus is not present in swabs taken from the readily accessible nasal cavity or throat as judged by the gold‐standard PCR assay, although there is some evidence that the viral antigen may still be present in the intestinal microbiome based on fecal assays.^4^ Social media has been used to report and document a “LONG COVID” support group (Claire Hastie on Facebook with 22 000 followers) as well as a phone app to track self‐reported symptoms of “LONG COVID”.^9^ A recent unreferred paper reported 205 symptoms submitted by 3500 people with mild to incapacitating aliments that can be from most organs in the body. This number of self‐reported symptoms is unlikely due to systematic selection bias and the lack of a control group who were not infected with the SARS‐CoV‐2 virus.^10^ The general consensus from population‐based UK studies ranges from 2.2% to 13.7% in female sex, middle age, and white ethnicity.^9^ These patients may have experienced mild, moderate or severe acute aliments in weeks 1–4. Figure 1 is a schematic representation of the timescale of infection and recovery in these patients, modified from Ayres.^11^ Often there are two or more aliments such as muscle fatigue, breathlessness, heart arrhythmias, joint pain, cough, hair loss, inflamed toes, anxiety, depression, cognitive disturbances (“brain fog”), thus being described as “from top to toe”.^2^ Initially, in mid‐2020 when anecdotal reports came from 10%–30% of adult patients who had a positive PCR test, they were dismissed as psychosomatic symptoms or a post‐viral syndrome that occurred with other viral illnesses such as mononucleosis.^4^ It is difficult to evaluate large numbers of anecdotal reports particularly from the lay press and social media as emphasized by Amin‐Chowdhury and Ladhani.^10^ More relevant for our review are prospective clinical studies. For instance, Darley et al.^13^ described the ADAPT study that was done over 12 months in a single institution with a carefully selected and defined group of 78 patients documented to have been infected with the virus. By following the ADAPT study, these workers subsequently characterized immunological profiles in 62 “LONG COVID” participants with confirmed SARS‐CoV‐2 infection and measured 29 analytes over 8 months.^14^ They found several cytokines mostly from interferon I and III classes that were highly elevated and stayed up over 8 months. They concluded that there was an elevated diffuse inflammatory cytokine profile in symptomatic “LONG COVID” subjects that was not observed in other subjects with post‐COVID‐19 recovery. Immune cell phenotyping also supported long‐lasting inflammation indicating that this may be driving symptomology of “LONG COVID.” Another prospective study reported a group of 134 patients (median age of 58) with positive RT‐PCR assays and radiological evidence of COVID‐19 pneumonia who were followed up to a median of 113 days post‐discharge primarily with validated questionnaires to record symptom burden.^12^ The only biochemical markers recorded were C‐reactive protein and white cell counts. These investigators identified three symptom clusters using a co‐occurrence matrix with Cluster A including myalgia and fatigue; Cluster B including low mood, anxiety, and sleep disturbance; and Cluster C comprising memory impairment, attention deficit, and cognitive impairment. Females were significantly higher in Cluster A but not significantly different in Clusters B and C. The authors doubted that “LONG COVID” had a distinct pathophysiology and that it was akin to post‐traumatic syndromes seen in the Gulf War Illness and post 9/11 syndrome that had a similar pattern of both physical and psychological symptoms.^12^ Another prospective study of 312 patients with mild COVID‐19 was done in Bergen, Norway and were followed for 6 months.^15^ 52% of a group of 61 home‐isolated young adults, aged 16–30 years, had symptoms at 6 months of loss of taste or smell, fatigue, dyspnea, impaired concentration, and memory problems which was a concern as it could interfere with their learning and study. It is hoped that these “LONG COVID” symptoms would encourage mass vaccination in the young.^15^

In the future, people with “LONG COVID” may be identified using “wearable” electronic devices such as a Fitbit™ wrist recorder that gives a real‐time quantitative measurement of mean resting heart rate (RHR) as demonstrated by Radin et al.^16^ They studied 234 individuals who had a positive PCR test for SAR‐CoV‐2 infection and compared them with 641 individuals who had a negative test. COVID‐19‐positive individuals experienced a transient bradycardia followed by prolonged tachycardia that did not return to baseline until approximately 12 weeks after symptom onset. This change in RHR was not seen in the SARS‐CoV‐2‐negative group. Interestingly, the wearable recorder identified a “LONG COVID” cohort (*n* = 32) who had >5 beats/min greater than their resting mean heart rate for more than 133 days who also self‐reported symptoms of cough, body ache, and shortness of breath, whereas 103 SARS‐CoV‐2‐infected patients had <1 beat/min elevated RHR over this period as did the SARS‐CoV‐2‐negative group.

## 4 WHAT COULD BE THE PATHOPHYSIOLOGICAL BASIS OF “LONG COVID”?

On the one hand, the multitude of organs that can be responsible for “LONG COVID” appears puzzling (reviewed by Nalbandian et al.^17^). On the other hand, Libby and Lüscher^18^ point out that COVID‐19 is an endothelial disease and all organs are perfused by the vascular microcirculation with capillaries composed of endothelium and pericytes. Interestingly, both cell types have been found to express the ACE‐2 protein on their cell membrane and SARS‐CoV‐2 infection has been widely reported to injure vascular walls and also cause blood clots in large and small blood vessels both in the periphery and brain.^19^, ^20^, ^21^ As reviewed by Østergaard,^22^ SARS‐CoV‐2 particles have been observed by electron microscopy in the endothelium of the lung, heart, kidney, brain, and skin of patients diagnosed with COVID‐19 and this is associated with morphological changes such as swelling, protrusion into the capillary lumen, and some endothelial cells undergoing apoptosis—findings indicative of severe hypoxia in surrounding tissues. Thus, endothelial damage is likely to disturb capillary flow pattern and apoptosis can impair signaling between intercellular connexin channels and upstream vascular smooth muscle cells. Furthermore, the luminal surface of the capillary endothelium is covered by a glycocalyx matrix that acts as a fluid barrier but elevated blood levels of tumor necrosis factor‐α in COVID‐19 would cause glycocalyx shedding. Shedding of glycocalyx causes profound changes in microvascular resistance and capillary hemodynamics. Østergaard concluded in his review that capillary damage from inflammation in COVID‐19 may contribute to both acute and long‐term symptoms of tissue hypoxia.^22^ In other words, if a “LONG COVID” person has breathlessness in the absence of exertion, is that due to capillary dysfunction in respiratory muscles? More seriously, this symptom may be due to lung or cardiac fibrosis. Or if angina is felt in the absence of exertion, is that due to capillary dysfunction in the heart? Or is “brain fog” due to capillary dysfunction impairing blood flow in cortical brain regions? Recent findings of SARS‐CoV‐2‐infected astrocytes throughout the brain could explain neurological symptoms of anxiety, depression, and “brain fog” that has impaired the transfer of glucose and lactate from astrocytes to neurons and does not need to be based on neuronal degeneration^23^ while SARS‐CoV‐2 infection of pericytes could be the basis of capillary constriction. In the case of the loss of smell, there is another mechanism based on ACE‐2 localization in the olfactory epithelial sustentacular cells but not in olfactory sensory neurons nor olfactory bulb neurons so the virus enters these glial cells and disrupts the normal supply of biochemicals to olfactory neurons.^24^ Furthermore, viral damage to the astrocytes could lead to disruption of the blood–brain barrier, entry of pro‐inflammatory cytokines from the systemic “cytokine storm,” neuro‐inflammation, and microglial activation.^25^ This scenario could trigger an increased incidence of neurological disorders such as depression, anxiety, and multiple sclerosis that has been reported in COVID‐19 patients.^25^ Molecular biology techniques using network‐based multimodal ‐omics analytic methodology for patients with COVID‐19 and Alzheimer's disease‐like pathology showed “scant evidence of direct brain and neuron damage by COVID‐19, but robust evidence for involvement of neuroinflammation and brain microvascular injury in COVID‐19”.^26^

## 5 HYPOTHESIS

**FIGURE 2:**
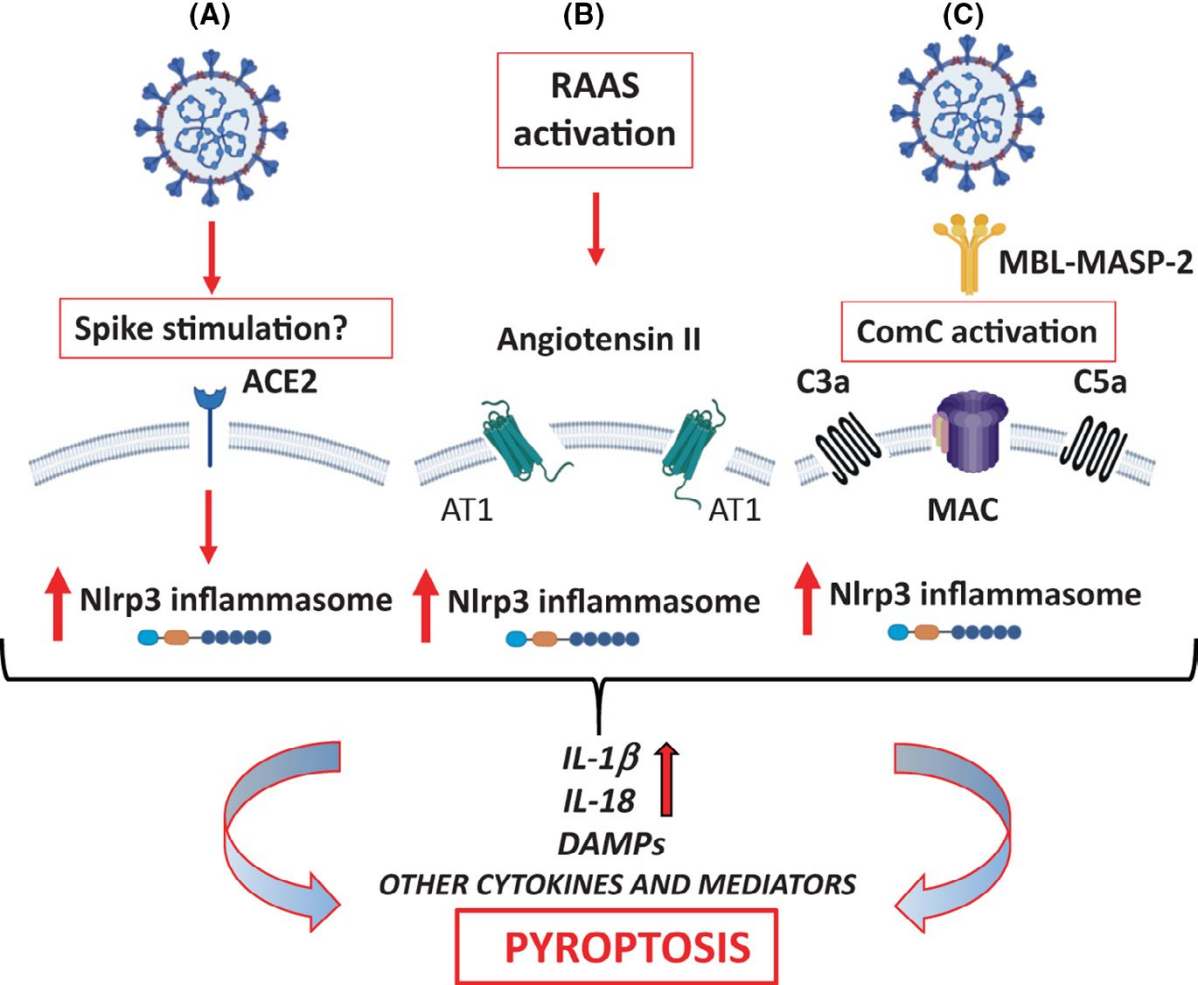
The pathways of Nlrp3 inflammasome activation in response to SARS‐CoV‐2 infection that may lead to initiation of cytokine storm and pyroptosis in cells. Reproduced with permission from Ratajczak and Kucia^29^

In order to replicate, viruses need to enter the cells of a host and alter its normal biochemical processes in order to synthesize viral proteins and nucleic acids. This modulates the normal intracellular redox state causing oxidative stress that is mediated by production of reactive oxygen species (ROS) and a subsequent decrease in glutathione, the main intracellular antioxidant. Furthermore, the overproduction of ROS causes mitochondrial dysfunction and DNA damage in the infected cell which inhibits the expression of the key redox‐sensitive transcription factor, nuclear factor, erythroid 2 like 2 (NRF2) which normally provides the primary cellular defenses against oxidative stress.^27^, ^28^ These conditions cause viral‐induced inflammation and often lysis/death of the infected cell due to activation of inflammasomes such as NLRP3 which are responsible for innate immunity in macrophages and epithelial cells. Research with the SARS‐CoV‐2 virus has shown that after it enters cells expressing the ACE‐2 cell surface enzyme by binding to it with its spike protein, it hijacks mitochondrial function causing increased mitochondrial ROS and activation of the intracellular proinflammatory NLRP3 inflammasome sensor since ACE‐2 is also a signaling receptor.^5^, ^29^ This leads to release of a storm of proinflammatory cytokines via caspase‐1 (such as interleukin‐Iβ and interleukin‐18) and danger‐associated molecular pattern molecules that amplify the innate immune response and leads to cell death by pyroptosis (Figure 2). Alternately or additionally, the loss of ACE‐2 enzyme activity and reduced synthesis of angiotensin 1–7 causes elevated levels of angiotensin 1–8 that binds to AT1 receptors on cells and this is also known to activate the NLRP3 inflammasome to cause cell death by pyroptosis in lung epithelium cells, endothelium, and cardiomyocytes.^30^, ^31^ These important findings of NLRP3 inflammasome and renin–angiotensin system (RAS) activation give a molecular and cellular explanation for the proinflammatory cytokine storm and acute multi‐organ failures widely reported in patients with severe COVID‐19 infection as well as future directions for discovering drug candidates to block activation of the NLRP3 inflammasome (reviewed by Lumbers et al.^32^). But do they explain “LONG COVID” and suggest drug treatments, appropriate doses, and administration timing for this puzzling disorder?

The key question is what is the molecular basis of the altered endothelium or pericytes or astrocytes? The most likely basis is oxidative stress due to viral alterations to the characteristic genotypic expression of those cells that impairs the expression of cytoprotective enzymes and proteins under the control of the antioxidative response element in the DNA. Normally, this element responds to the key redox‐sensitive transcription factor, NRF2 by increasing the rate of transcription of cytoprotective genes for antioxidant enzymes such as glutamate–cysteine–ligase that then increases the synthesis of the tripeptide glutathione which is the key intracellular scavenger of ROS.^33^ In addition, NRF2 also suppresses Nuclear Factor‐kappa B (NF‐kB), a transcription factor involved in inflammatory responses and downregulates the NLRP3 inflammasome.^34^, ^35^ NRF2 also provides substrates for mitochondria thus increasing ATP production in cells. Studies with Vero cells infected with the Enterovirus 71, a single positive RNA strand virus, showed a reduced expression of the NRF2 protein and an enhanced expression of the Keap1 protein that normally binds to NRF2 thus acting as a repressor of the NRF2 transcription factor which contributes to increased ROS generation and cell death.^36^

## 6 POTENTIAL PHARMACOLOGICAL TREATMENTS FOR “LONG COVID”

While there are almost no drugs that have been found to minimize the symptoms of “LONG COVID,” there are demands by patients for drugs to treat their many symptoms. Drug development is a very slow process with generally 10+ years elapsing between identifying a promising candidate in preclinical animal models and taking that through to an approved drug. It is generally accepted that one useful strategy is to explore the ability of existing approved drugs to treat patients with COVID‐19 disease and its complications such as “LONG COVID”—an approach referred to as treating the host.^37^, ^38^

However, this approach requires a thorough analysis of the pathophysiology of the disease, the dose–response curve in humans for the specific decision, and a clinical understanding of the patient group to be treated. As chronic inflammation due to elevated proinflammatory cytokines have been identified in “LONG COVID,” anti‐inflammatory drugs could be considered. However, the cytokine activity could be cause or effect of the problem, and thus consideration of a therapeutic strategy needs to be scrutinized; the drug development world is littered with failed examples of drugs been used to treat a single marker noted in a disease population.^39^

## 7 THERAPEUTIC OPTIONS FOR ACTIVATING NRF2 IN “LONG COVID” SUBJECTS

Oxidative stress in endothelial cells is established as a pathological mechanism in diabetes which precedes the development of diabetes‐associated vascular complications and it reduces the bioavailability of the potent endothelial mediator, nitric oxide.^40^ Furthermore, these authors showed that oral treatment of the Akita mouse model of diabetes with a NRF2 activator, dh404 (3 mg/kg daily), upregulated NRF2‐responsive genes and cytoprotective enzymes and improved vascular relaxation of aortic rings in vitro. Similarly, during normal aging in rats, arteries taken from 20‐month‐old rats exhibit oxidative stress and no longer respond to the drug carbachol by relaxing, whereas the same arteries from 3‐month‐old rats relax upon addition of carbachol.^41^ However when isolated coronary arteries from 20‐month‐old rats were incubated with a known NRF2 activator, sulforaphane for 1 h then this significantly improved endothelium‐dependent relaxation to carbachol. Immunofluorescence studies with antisera confirmed that sulforaphane incubation resulted in a significant expression of NRF2 protein in isolated coronary arteries. Also, oltipraz, another known NRF2 activator, fully restored endothelium‐dependent relaxation in coronary arteries after a 1‐h incubation.^43^ Another study of changes in NRF2 with ageing^42^ was done using cultured primary bronchial epithelial cells prepared from young adult males (28–29 years) and old adults (67–69 years).^42^ It was found that incubation of cells with sulforaphane (2.5 µM for 12 h) resulted in significant inducibility of representative NRF2‐regulated antioxidant genes (NADH Quinone Oxidoreductase‐1; glutamate cysteine ligase subunits) as measured by mRNA expression but that the young adults gave values approximately three times higher than the older adults. The authors concluded that NRF2 signaling declined with age and this may be due to inducible expression of two NRF2 suppressor proteins c‐Myc and Bach‐1 which were increased in the older donor bronchial epithelial cells compared to that from young adults.^42^ They also suggested that while the elderly have a basal capacity to cope with oxidation, they are less able to increase the antioxidant enzymes in response to stress. Therefore, therapeutic approaches based on activating NRF2 to deal with oxidative stress may not be as effective in the elderly as in young adults.^42^

Since oxidative stress is the earliest molecular disturbance in viral‐infected cells such as endothelial cells which then causes capillary damage and local hypoxia, there is a compelling case to trial NRF2 activators to enhance gene expression leading to transcription of enzymes to elevate the intracellular antioxidant, glutathione. While at least 10 distinct classes of NRF2 activators have been characterized over the last 16 years,^43^ many of these chemicals are of plant origin such as polyphenols, isothiocyanates, and flavonoids that often have poor drug candidate features. The exception as a drug to activate NRF2 could be melatonin.^44^, ^45^

### 7.1 Melatonin

**FIGURE 3:**
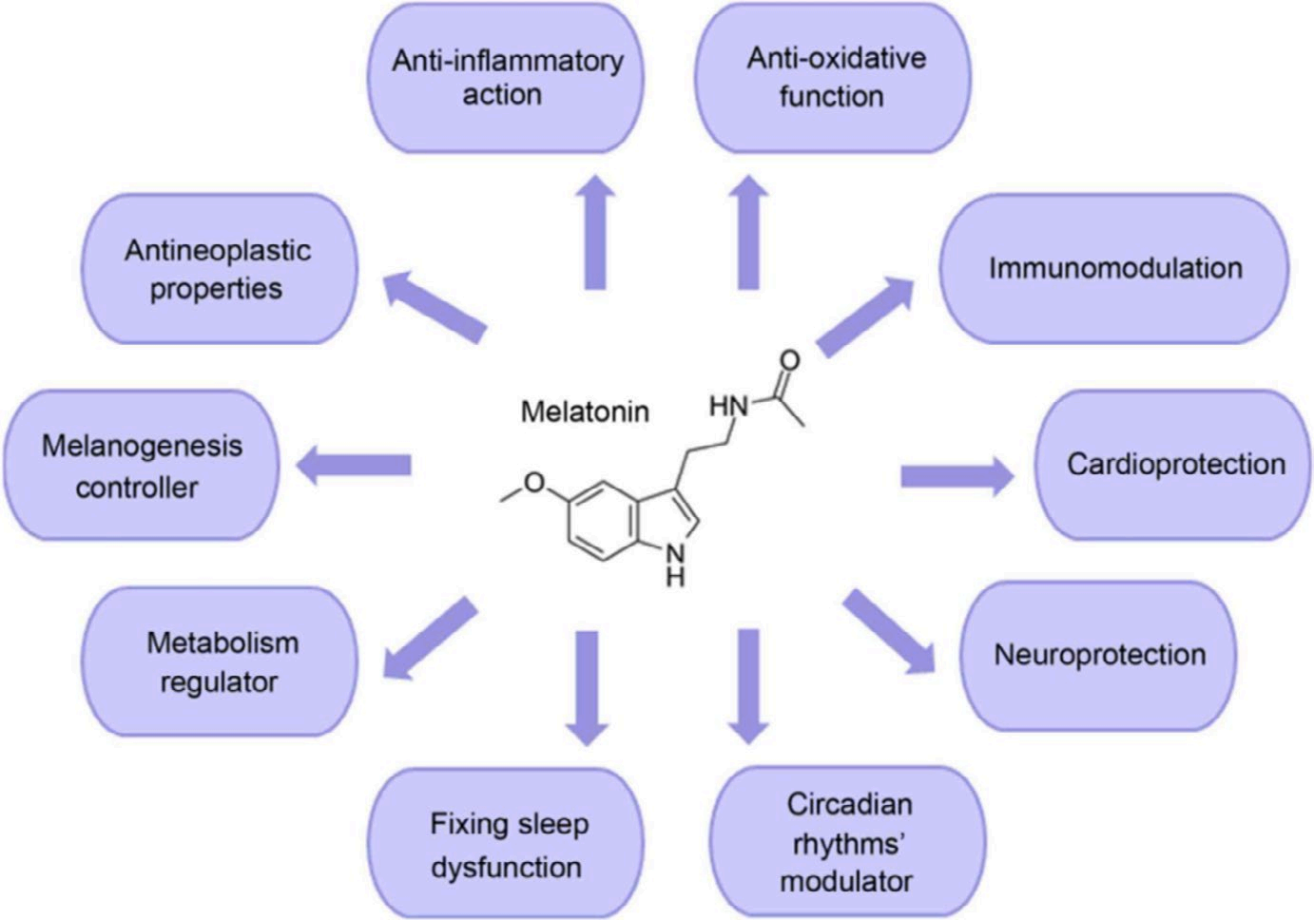
Mechanisms of action of melatonin—reproduced with permission from Vlachou et al^54^

Melatonin was originally identified as a hormone released from the pineal gland at night but not during the day and was known as the “dark hormone” that promoted sleep.^46^ Melatonin is now known to be synthesized and released, but not in a circadian rhythm, from other tissues such as retina, bone marrow, gastrointestinal tract, and placenta.^47^, ^48^, ^49^ When ingested by humans, it is regarded as having a good safety profile established over the last 50 years.^50^, ^51^ More recently, it was found to be an NRF2 activator/antioxidant that exerts neuroprotection in animal models.^52^, ^53^, ^54^ Prior to COVID‐19 in 2020, melatonin has been studied in bacterial sepsis, both in vitro and in vivo.^55^, ^56^, ^57^, ^58^, ^59^ Sepsis has a high mortality due to multiple organ failure, massive cytokine release, and oxidative stress due to mitochondrial malfunction driving systemic inflammation.^60^ Galley's laboratory found that antioxidants targeted to mitochondria such as melatonin reduced organ damage in rats in vivo.^57^ With in vitro models of sepsis, both melatonin and its 6‐hydroxy metabolite reduced the levels of the inflammatory cytokine IL‐6, decreased oxidative stress as well as NFkB activation, and improved mitochondrial function.^56^ It was suggested that 6‐hydroxymetabolite would contribute to the antioxidant effect of melatonin when high doses of melatonin were given in vivo. A phase I study in human volunteers showed no adverse effects after oral melatonin (20, 30, 50, or 100 mg) and melatonin was cleared with a half‐life of 52 min across all doses.^58^ While its oral bioavailability is poor (~15%),^61^ recently it has been suggested that this could be improved using “smart nanocarriers” that might enhance delivery of melatonin to mitochondria to reduce organ damage.^62^ It has been suggested that melatonin, in addition to NRF2 activation, may have additional mechanisms of action,^54^ see Figure 3. Also, Cardinali et al.^44^ suggested that melatonin acts via activation of sirtuin‐1 to inhibit polarization of macrophages from a pro‐inflammatory type, thereby reducing cytokines such as TNF‐α, IL‐1β, and IL‐6 to an anti‐inflammatory subtype that produces IL‐10.

Melatonin has been reviewed recently as an early drug treatment for acute COVID‐19.^54^, ^63^ However, it could also have a role in treating “LONG COVID” patients who experience neuropsychiatric symptoms such as insomnia, depression, and anxiety. Melatonin is accepted as safe for treating insomnia in the elderly^64^, ^66^ and sold in pharmacies in Australia and UK as a 2 mg slow release tablet (Circadin™).^64^ But it is not an acute hypnotic agent and generally needs to be taken for a month or two to re‐set the desired night–day circadian rhythm. In the United Kingdom, a clinical trial protocol has been published for evaluating Circadin™ 6mg for reducing sleep disturbances in doctors and nurses working night‐shifts in hospitals.^65^ This pilot phase II feasibility trial showed that Circadin™ 6 mg was safe for staff with no concerning side effects and that a larger definitive study (such as one including “LONG COVID” subjects experiencing sleep disturbances) would be safe. Melatonin can be taken during the day without causing daytime sleepiness^66^ which makes it feasible for treating other brain disorders in addition to insomnia. For example, in depression, alterations in both the sleep–wake cycle and daily rhythms in secretion of hormones such as cortisol are common. Exogenous melatonin could have some effect in restoring these rhythms.^67^, ^68^

Furthermore, melatonin has been shown to relieve a depressed behavioral state in mice as well as stimulating neurogenesis in the hippocampus, a neurobiological response to clinically effective antidepressant drugs.^69^, ^70^ Other issues for clinical trials include dosing regimen^66^ in view of the short elimination half‐life of melatonin^58^, ^61^ and concomitant symptoms. The melatonin receptor agonist agomelatine^67^ which is already approved for treating depression could be trialed in “LONG COVID” patients who are experiencing depression. As more knowledge about the biology of “LONG COVID” comes available, the optimal hypothesis for trial testing, choice of drug therapy and dosing regimen, and clinical outcomes of interest will become more clear, in order to formulate a clinical trial.

## 8 FUTURE CLINICAL TRIALS

To summarize “LONG COVID,” it is a multi‐organ/multi‐system disorder that follows on from an acute infection with the SARS‐CoV‐2 virus with an unpredictable relapsing‐remitting response that occurs for weeks to months later. In the United Kingdom, over 1 million people are estimated to be living with “LONG COVID” for at least 6 months.^71^ Unfortunately, there is no diagnostic or imaging modality to test these people and follow the time course of the disorder. Nor will there be found a single drug to relieve the symptoms. Thus, clinical trials with different drugs are needed to assess their efficacy for treating “LONG COVID.” In the UK, a multi‐center trial called HEAL‐COVID^72^ has been set up with its aim “Helping Alleviate the Longer‐term consequences of COVID‐19.” It is run from 102 sites in the UK with Research Nurses who recruit people when discharged from hospitals after acute treatment for COVID‐19. They will be randomly allocated to receive either Atorvastatin (40 mg daily for 12 months) or Apixaban (2.5 mg twice daily for 2 weeks) or the usual standard care offered by that hospital upon discharge. They will be surveyed weekly via an app on a smartphone or a phone call from the Research Nurse with questions on their symptoms, quality of life, and experience of the trial.

Atorvastatin is a good choice as it has pleiotropic actions that improve endothelial function by decreasing oxidative stress and vascular inflammation.^73^ Apixaban is being trialed as a prophylactic anticoagulant against venous thromboembolism since COVID‐19 is associated with an increased risk of thromboembolism and endothelial damage. However, as pointed out by Chandra et al.^74^ a vast majority of patients present with undiagnosed thromboembolism following COVID‐19 but there has not been a randomized clinical trial completed to determine the optimal anticoagulant, dosage, and duration of anticoagulation in these patients. Another drug worthy of study on “LONG COVID” patients is an angiotensin receptor blocker such as Telmisartan. As reviewed by Cooper et al.,^75^ “LONG COVID” patients show numerous cardiovascular complications such as myocarditis, microvascular angina, cardiac arrhythmias, and blood pressure abnormalities due to dysregulation of the renin–angiotensin–aldosterone System primarily by angiotensin II and that antagonists such as Telmisartan could re‐establish cardiovascular homeostasis by blocking the actions of angiotensin II on AT1 receptors.

## DISCLOSURE

None of the authors have any conflicts of interest. No grants were received to fund this publication. No Ethics Approval was required for this publication. No patient Consent was required. No clinical trial registration was required.

## ETHICAL APPROVAL

Not applicable as no animal or human experimentation was carried out for this review.

## AUTHOR CONTRIBUTIONS

Bevyn Jarrott drafted the manuscript with substantial input from Jennifer H. Martin and Richard Head. All authors made significant contributions to the conception and design of the manuscript, revised it critically for intellectual context and gave final approval of the final version.

## NOMENCLATURE OF TARGETS AND LIGANDS

Key protein targets and ligands in this article are hyperlinked to corresponding entries in http://www.guidetopharmacology.org, the common portal for data from the IUPHAR/BPS Guide to PHARMACOLOGY,^76^ and are permanently archived in the Concise Guide to PHARMACOLOGY 2019/20.^77^

## Data Availability

The data that support the findings of this study are available from the corresponding author upon request.
